# A deep learning-based radiomics approach to predict head and neck tumor regression for adaptive radiotherapy

**DOI:** 10.1038/s41598-022-12170-z

**Published:** 2022-05-27

**Authors:** Shohei Tanaka, Noriyuki Kadoya, Yuto Sugai, Mariko Umeda, Miyu Ishizawa, Yoshiyuki Katsuta, Kengo Ito, Ken Takeda, Keiichi Jingu

**Affiliations:** 1 Department of Radiation Oncology, Tohoku University Graduate School of Medicine, 1-1 Seiryo-machi, Aoba-ku, Sendai, 980-8574 Japan; 2 Department of Radiological Technology, School of Health Sciences, Faculty of Medicine, Tohoku University, Sendai, Japan

**Keywords:** Head and neck cancer, Biomedical engineering

## Abstract

Early regression—the regression in tumor volume during the initial phase of radiotherapy (approximately 2 weeks after treatment initiation)—is a common occurrence during radiotherapy. This rapid radiation-induced tumor regression may alter target coordinates, necessitating adaptive radiotherapy (ART). We developed a deep learning-based radiomics (DLR) approach to predict early head and neck tumor regression and thereby facilitate ART. Primary gross tumor volume (GTVp) was monitored in 96 patients and nodal GTV (GTVn) in 79 patients during treatment. All patients underwent two computed tomography (CT) scans: one before the start of radiotherapy for initial planning and one during radiotherapy for boost planning. Patients were assigned to regression and nonregression groups according to their median tumor regression rate (ΔGTV/treatment day from initial to boost CT scan). We input a GTV image into the convolutional neural network model, which was pretrained using natural image datasets, via transfer learning. The deep features were extracted from the last fully connected layer. To clarify the prognostic power of the deep features, machine learning models were trained. The models then predicted the regression and nonregression of GTVp and GTVn and evaluated the predictive performance by 0.632 + bootstrap area under the curve (AUC). Predictive performance for GTVp regression was highest using the InceptionResNetv2 model (mean AUC = 0.75) and that for GTVn was highest using NASNetLarge (mean AUC = 0.73). Both models outperformed the handcrafted radiomics features (mean AUC = 0.63 for GTVp and 0.61 for GTVn) or clinical factors (0.64 and 0.67, respectively). DLR may facilitate ART for improved radiation side-effects and target coverage.

## Introduction

The clinical success of radiotherapy for cancer depends on precise targeting of radiation to tumor tissue while minimizing exposure to healthy noncancerous tissue. However, the anatomic coordinates of the tumor may change during treatment due to regression, necessitating re-evaluation of dose distribution, termed adaptive radiotherapy (ART). For radiotherapy of head and neck cancers, complex dose distribution regimens such as intensity-modulated radiotherapy (IMRT) may still expose peritumoral organs at risk (OARs) due to anatomical changes such as reduction in tumor volume^1^. These changes in head and neck tumor volume may also substantially reduce the minimum dose within the target^2^. Regression of the clinical target volume (CTV) is particularly rapid during the first two weeks of radiotherapy^2^, so the impact on dose distribution may be particularly great during this early treatment stage. To improve tumor targeting and reduce OAR exposure, various ART protocols have been developed, in which radiotherapy is re-planned according to anatomical changes and tumor shrinkage, with documented efficacy for head and neck cancer^1–4^. With the recent widespread use of magnetic resonance imaging (MRI)-guided linear accelerators, it is conceivable that in the near future, patients with head, and neck cancer may be treated using online ART protocols revised regularly based on current tumor anatomy. However, online ART (especially adaptation to shape) is both labor intensive and costly as it involves regular rescanning, recontouring, replanning, and plan verification. Therefore, we speculated that if we could distinguish patients likely or unlikely to demonstrate early radiation-induced tumor regression before starting radiotherapy, it may be possible to schedule frequent online ART only for early “regressors” and use a more fixed protocol with less frequent adjustment for early “nonregressors.”

Several previous studies have attempted to predict ART requirement before starting radiotherapy. Surucu et al. predicted tumor shrinkage using a decision tree algorithm consisting only of clinical factors^5^, while several other studies used features manually extracted from medical images as predictive biomarkers (referred to as handcrafted radiomics features)^6–8^. Yu et al. and Alves et al. suggested that specific handcrafted radiomics features can be predictive of ART eligibility among patients with head and neck cancer based on the notion that some of these features reliably predict tumor regression^9,10^. Although these studies distinguished ART and non-ART groups, they did not directly predict tumor regression in head and neck cancer patients. In general, handcrafted radiomics features are limited to anatomical (e.g., tumor size, shape, volume, and position), intensity (first-order), and texture (second-order) characteristics. Thus, we speculate that reliable prediction of tumor regression will require the integration of more higher-order features.

Deep learning using convolutional neural networks (CNNs) offer great potential for improving medical imaging applications, such as object detection^11^, classification^12^, segmentation^13^, regression prediction^14^, and error detection^15^, as well as dose distribution planning for radiotherapy^16,17^. Transfer learning in pretrained CNNs is widely used for applications where the number of patients is insufficient for conventional deep learning. The core feature extraction method in transfer learning is to freeze all CNN layers pretrained on a larger external dataset to act as a fixed feature extractor for new inputs like medical images^18^. Such transfer learning has demonstrated potential for the prognosis^19^, metastasis prediction^20^, and differentiation of benign from malignant nodules^21^. CNNs trained on large datasets (e.g., natural images) have already learned the regularity of various objects; as a result, the deep features extracted can reflect higher-order patterns and capture more image heterogeneity^19^. Transfer learning as a feature extraction method thus has the potential to provide more information than handcrafted radiomics features for predicting tumor behavior.

Although deep feature extraction by pretrained CNNs has achieved prediction accuracy exceeding that of handcrafted radiomics features and clinical factors^22,23^, it has not yet been used to predict tumor regression in head and neck cancer. In this study, we propose a deep learning-based radiomics (DLR) approach for adaptive radiotherapy to predict early radiotherapy-induced primary gross tumor volume (GTVp) regression and nodal gross tumor volume (GTVn) regression before treatment onset.

In this study, we first compared the predictive performance of our proposed DLR approach to previously reported models incorporating clinical factors and handcrafted radiomics features. We then comprehensively evaluated multiple deep learning models for extracting deep features using various feature selection and machine learning algorithms to identify those with high predictive performance for tumor regression. Briefly, this study used GTVp and GTVn images as inputs to deep learning models pretrained on a larger set of natural images, and extracted deep features from the hidden layers to predict GTVp and GTVn regression versus nonregression after a median of 15 radiotherapy applications (range, 11–20).

## Materials and methods

### Patient characteristics

Patients who received chemoradiotherapy or radiotherapy to the head and neck region at Tohoku University Hospital were retrospectively enrolled as study candidates. Participants were then selected according to inclusion and exclusion criteria (below) as shown diagrammatically in Supplementary Fig. 1. A total of 255 patients were excluded according to the inclusion and exclusion criteria, and finally 96 patients were enrolled for GTVp monitoring and 79 for GTVn monitoring. Patients were excluded for the following reasons: treatment with three-dimensional (3D) conformal radiotherapy (n = 10), no boost computed tomography (CT) or no GTV recontouring on boost CT (n = 47), primary tumor that was not head and neck cancer (n = 6), neither GTVp nor GTVn following surgery (n = 23), treatment with intra-arterial injection chemotherapy (n = 4), and severe image artifacts (n = 1). Patients with tumors < 5 cm^3^ were also excluded (GTVp: n = 33, GTVn: n = 53) because a previous study reported that small volumetric changes benefit less from ART^4^ and the image characteristics such as texture information extracted from tumors < 5 cm^3^ are limited^24^. All segmentations were assessed by a medical physicist for initial and boost CT. Patients with large differences in contouring between the initial and boost CTs were also excluded (inadequate contouring: n = 2). For these two patients, the area contoured as the CTV in the initial CT was broadly contoured as the GTV in the boost CT. The characteristics of the selected patients are summarized in Supplementary Tables 1 and 2. Tumor sites were nasopharynx, oropharynx, hypopharynx, oral cavity, larynx, and paranasal sinus.

All patients were treated with radiation therapy for purposes of radical or postoperative recurrence. The research design, data collection and management protocols, and scientific rationale of this study were approved by the Ethics Committee of Tohoku University Hospital. In addition, all experiments were performed in accordance with relevant institutional and national guidelines and regulations. Given the retrospective nature of this study and the fact that no samples were obtained from human bodies, the requirement for informed consent was waived by the Ethics Committee of Tohoku University Hospital.

### CT image acquisition

Patients were prescribed 44 Gy/22 fraction (fr) (or 40 Gy/20 fr) in the region of CTV primary, CTV nodal, and CTV prophylactic with 5 mm added to the planning target volume (PTV) margin as initial treatment, and 26 Gy/13 fr (or 30 Gy/15 fr) in the region of CTV primary and CTV nodal with 5 mm added to the PTV margin as boost treatment. In our hospital, a two-step method is adopted in which the patient is scanned again during radiotherapy, and a boost plan is created based on the rescanned CT image. In other words, the patient receives two CT scans, one before the start of radiotherapy for the initial plan and one during radiotherapy for the boost plan. All CT scans were acquired using the SIEMENSE SOMATOM Definition AS + system with pixel size of 1.17–1.27 mm and a slice thickness of 2–2.5 mm.

### Classification of tumor regression and nonregression groups

The GTVp and GTVn were manually contoured by experienced radiologists on both the initial planning and boost CT images. The relative volume changes (ΔGTVp and ΔGTVn) were calculated by subtracting the boost CT volume from the initial CT volume and then dividing it by the initial CT volume. The period between the first CT scan and boost CT scan was different for all patients. To eliminate any effect caused by the gap between the first and second CT scans, the volume change rates were calculated by dividing ΔGTVp and ΔGTVn by the number of radiotherapy sessions received before the boost CT scan as follows (using ΔGTVp/treatment day as an example):

$$\Delta GTVp/treatment\;day = \frac{{{\text{Initial}}\;{\text{GTVp}}\;{\text{volume}} - {\text{Boost}}\;{\text{GTVp}}\;{\text{volume}}}}{{{\text{Initial}}\;{\text{GTVp}}\;{\text{volume}}}}/treatment\;day$$

**Figure 1:**
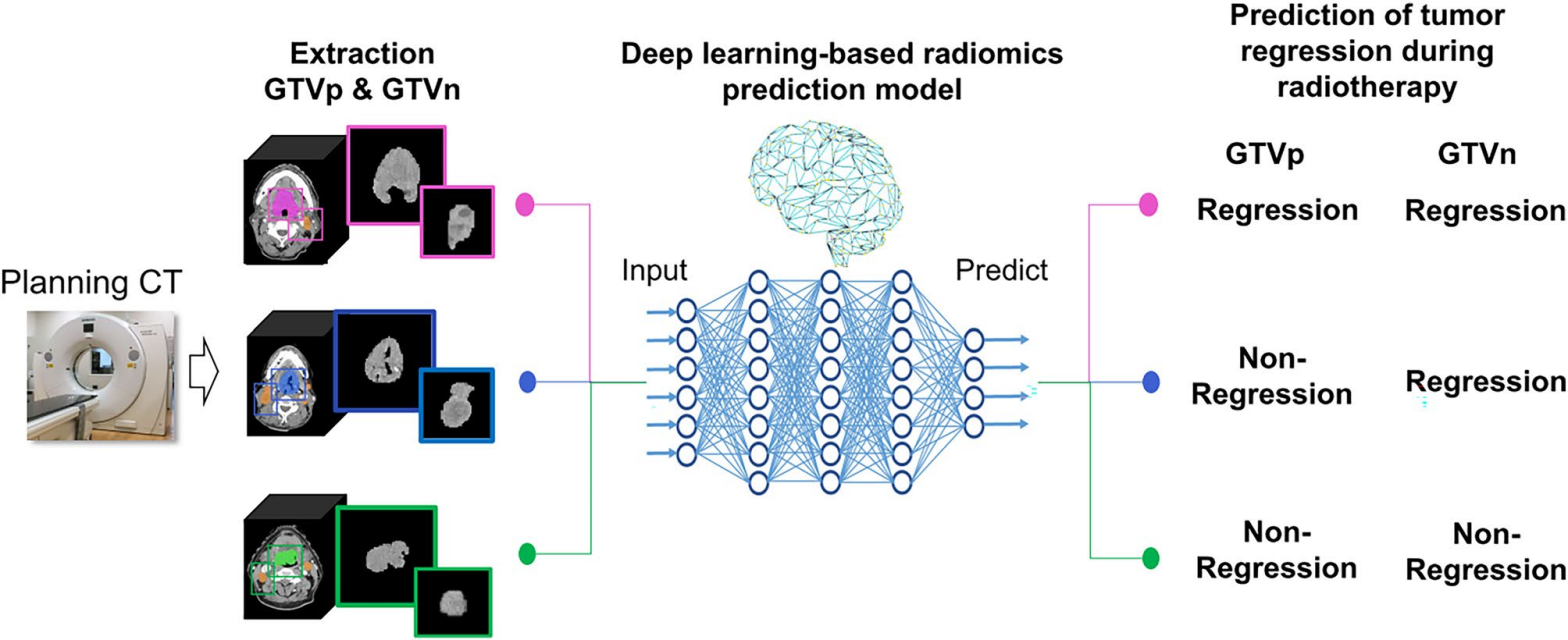
Schematic diagram of a deep learning-based radiomics approach for predicting early radiation-induced tumor regression utilizing only CT images of gross tumor volume (GTV) acquired before radiotherapy.

**Figure 2:**
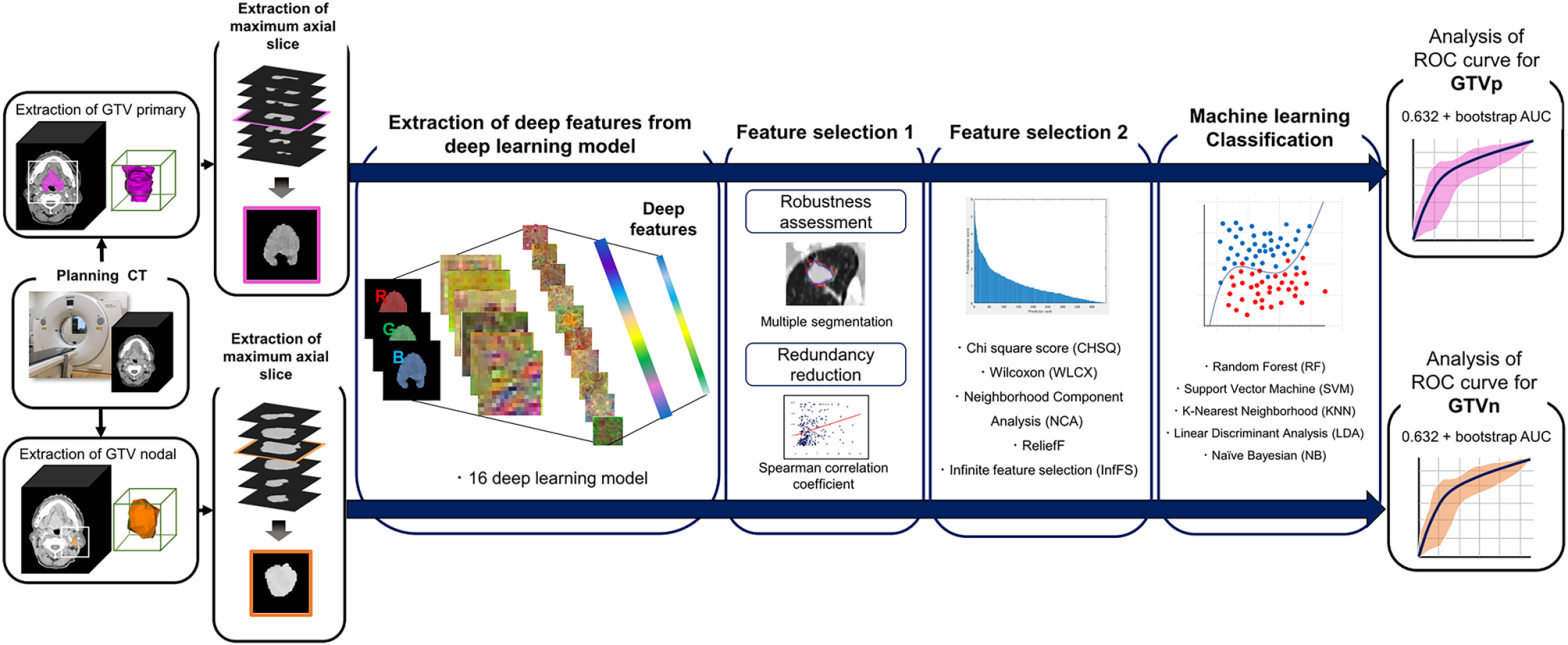
Workflow of the deep learning radiomics approach. Axial CT slices of primary gross tumor volume (GTVp) and nodal gross tumor volume (GTVn) were inputted to 16 convolutional neural network (CNN) deep learning models pretrained on natural images, and deep features were extracted. Next, the most robust features were selected and redundant features eliminated (selection step 1). Then, the top 10 features for each of the five feature selection algorithms were identified (selection step 2). Machine learning algorithms were used to predict primary GTV (GTVp) and nodal GTV (GTVn) regression versus nonregression. Finally, the predictive performance of each model was evaluated using the mean 0.632 + bootstrap area under the curve (AUC) method with 1000 iterations.

The median ΔGTVp/treatment day and median ΔGTVn/treatment day for all patients were used as thresholds to classify patients into tumor regression and nonregression groups. We then predicted these two classifications by the DLR approach. Figure 1 presents a schematic diagram of the general study workflow and Fig. 2 illustrates the detailed workflow of the DLR approach.

### DLR

#### Overview

We extracted one axial slice showing the maximum GTVp or GTVn cross-sectional area from the planning CT and used it as input to 16 CNN models pretrained on natural images. One thousand deep features were extracted from the hidden layer of each model, which were used to predict tumor regression. Next, feature selection was conducted in two steps. In the first (feature selection step 1), robust features were selected, and redundant features eliminated. In feature selection step 2, the top 10 features were selected by each of the five algorithm-based feature selection methods. Machine learning algorithms were then used to predict GTVp and GTVn regression and nonregression. In this study, 25 unique models were built for each CNN by combining the five algorithm-based feature selection methods and five machine learning algorithms. Finally, we evaluated the predictive performance of each model order using the mean 0.632 + bootstrap area under the curve (AUC) method with 1000 iterations.

#### Deep feature extraction

GTVp and GTVn were extracted from planning CT images. Feature values extracted from images with different voxel sizes show large variation^25^, so all CT images were first resampled to 1 × 1 × 1 mm^3^ using the nearest neighbor algorithm. One axial slice showing the maximum GTVp or GTVn cross-sectional area was identified, and an image of 100 mm × 100 mm was extracted centered on the tumor center of gravity. If there were several tumors in this identified maximum axial slice, the center of gravity of the tumor with largest area in the 100 mm × 100 mm image was identified, and a new 100 mm × 100 mm image was extracted centered on this largest tumor center of gravity. The 100 mm × 100 mm size was sufficient to include most of the tumor area in all cases. The non-GTV area was then set to the minimum CT value to extract only biomarkers (features) from within the tumor. The intensity of the image was modified using the window level (50 Hounsfield units [HU]) and window width (350 HU) for the abdominal condition to improve contrast within the tumor. Images used as inputs to deep learning networks are usually composed of three channels (Red, Green, Blue), and a previous report found that prediction accuracy improves when three channels are used as network input compared to only one^21^. Therefore, the single grayscale CT image was copied to produce three images as input. Finally, because each of the 16 deep learning models described below has a different input size, we resized the input images to fit each model using the bilinear interpolation algorithm. We used three channels of two-dimensional (2D) images as input for the deep learning networks because most CNN model layers pretrained on natural image datasets were constructed with three channels of 2D inputs. Image preprocessing was performed using MATLAB R2020b (MathWorks, Natick, MA, USA).

Due to the small patient sample, we extracted deep features using a method that freezes the weights of all CNN model layers already pretrained on many natural image datasets. We used the deep learning toolbox of MATLAB and downloaded 16 CNNs, SqueezeNet, GoogleNet, Inceptionv3, DenseNet201, MobileNetv2, ResNet18, ResNet50, ResNet101, Xception, InceptionResNetv2, ShuffleNet, NASNetMobile, NASNetLarge, DarkNet19, DarkNet53, and AlexNet, all pretrained on the ImageNet Large-Scale Visual Recognition Challenge (ILSVRC)^26^. We then extracted 1000 deep features from the last fully connected layer of each network. For three networks (SqueezeNet, DarkNet19, and DarkNet53) that did not have a fully connected layer at the end, 1000 deep features were extract from the layer before the last softmax layer. All deep features were normalized by z-score.

#### Feature selection

For handcrafted radiomics features, dimension reduction technique such as reproducibility analysis, collinearity analysis, and algorithm-based feature selection are used for feature selection^27^. Therefore, we also used these three feature selection methods to select deep features so that results are easily comparable to previous models based on handcrafted radiomics features.

Features extracted from medical images are susceptible to various sources of variability, such as respiratory motion^28^, multiple contouring^29^, and different CT protocols^30^. Therefore, we first used multiple segmentation to select the most robust deep features. First, CT images of 20 patients with lung cancer with nodule segmentation and Reference Image Database to Evaluate Therapy Response (RIDER) data were downloaded from The Cancer Imaging Archive online Quantitative Imaging Network multisite collection^31^. Nine segmentations were already delineated for one patient in this dataset. Because all 20 patients had nine segmentations, the deep features were extracted from a total of 180 segmentations. Robustness was evaluated using the intraclass correlation coefficient (ICC) for Case 3A^29,32^. This method evaluates the inter-observer variability of the segmentations. We used MATLAB as the analysis software. Features with ICC > 0.7 were selected as robust. In other words, deep features that fluctuated greatly in value due to slight differences in segmentation were deemed not sufficiently robust and excluded. In the next step, collinearity analysis was used to remove redundant features. If the Spearman’s correlation coefficient between any two features was > 0.8, then the mean correlations with all other features were calculated and the feature with the higher mean coefficient was eliminated from the pair, based on the method of Li et al.^33^. We applied this method to all feature pairs with Spearman’s correlation coefficient > 0.8.

As the last step, an algorithm-based feature selection was used. Multiple algorithm-based feature selection methods are available, and the final prediction accuracy is expected to vary depending on the choice of algorithm. Therefore, it is necessary to investigate different feature selection methods to optimized DLR model performance. In this study, five filter-type feature selection methods were used to rank the deep features: Chi square score (CHSQ), Wilcoxon (WLCX), Neighborhood Component Analysis (NCA), ReliefF, and Infinite Feature Selection (infFS)^34^. Finally, each selection method was used to select the top 10 features according to rank. The CHSQ, WLCX, NCA, and ReliefF methods were used, given that they are available in the Statistics and Machine Learning Toolbox of MATLAB and applicable to the binary classification problem. We also used infFS considering that it achieved the best performance in the PASCAL VOC 2007–2012 classification tasks^34^.

#### Machine learning prediction

Different internal algorithms for machine learning may demonstrate highly variable classification accuracies when provided with different sets of features. Thus, the results from a single machine learning algorithm may not be representative of the general predictive utility of a given deep feature or feature set. Therefore, this study used five machine learning algorithms to predict tumor regression and nonregression: Random Forest (RF), Support Vector Machine (SVM), K-nearest neighborhood (KNN), Naïve Bayses (NB), and Linear Discriminant Analysis (LDA). Detailed hyperparameter settings of the machine learning models are shown in Supplementary Table 3. We also used five algorithm-based feature selection methods, so 25 models in total were constructed for each CNN. We evaluated the predictive accuracy of each model in order to comprehensively evaluate the performance of specific deep features.

#### Evaluation

The 0.632 + bootstrap AUC metric with 1000 iterations was used to evaluate each model as this method has demonstrated lower variance, bias, and mean squared error for a small number of samples and a large number of features^35^. The 0.632 + bootstrap AUC metric was defined by.

$$\widehat{{AUC}}_{{0.632 + }} = \frac{1}{B}\mathop \sum \limits_{{b = 1}}^{B} \left[ {\left( {1 - a\left( b \right)} \right)AUC\left( {X,X} \right) + a\left( b \right)AUC'\left( {X^{{*b}} ,~X^{{*b}} \left( 0 \right)} \right)} \right],$$

where $${\text{AUC}}^{{\prime }} \left( {{\text{X}}^{{{\text{*b}}}} ,{\text{~X}}^{{{\text{*b}}}} \left( 0 \right)} \right) = max\left\{ {0.5,~{\text{AUC}}\left( {{\text{X}}^{{{\text{*b}}}} ,{\text{~X}}^{{{\text{*b}}}} \left( 0 \right)} \right)} \right\}$$, $$a\left( b \right) = \frac{0.632}{{1 - 0.368 \cdot R\left( b \right)}}$$, and

$$R\left( b \right) = \left\{ {\begin{array}{*{20}l} 1 \hfill & {{\text{If}}\;{\text{AUC}}\left( {{\text{X}}^{{\text{*b}}} ,{\text{ X}}^{{\text{*b}}} \left( 0 \right)} \right) \le 0.5} \hfill \\ {\frac{{AUC\left( {X,X} \right) - AUC\left( {X^{*b} , X^{*b} \left( 0 \right)} \right)}}{{AUC\left( {X,X} \right) - 0.5}}} \hfill & {{\text{If}}\;{\text{AUC}}\left( {{\text{X}},{\text{X}}} \right) > {\text{AUC}}\left( {{\text{X}}^{{\text{*b}}} ,{\text{ X}}^{{\text{*b}}} \left( 0 \right)} \right) > 0.5} \hfill \\ 0 \hfill & {{\text{otherwise}}} \hfill \\ \end{array} } \right.$$

If the patient sample is represented by **X**, and *X* represents the data vector, a sample of size N is represented by **X** = (*X*_1_, *X*_2_…, *X*_*N*_). The AUC (**X**, **X**) represents the AUC of training on patient sample **X** and testing on patient sample **X**. However, this causes a bias toward better AUC because the training and testing are on the same data set. X^*^ = (*X*_1_^*^, *X*_2_^*^…, *X*_N_^*^) represents a boot sample of size N that has been randomly extracted from data **X**. In this boot sample, some data vectors may not appear, while others may appear once, twice, or three times (etc.). B represents the number of the boot sample, X^*1^, X^*2^,…,X^*B^, where each boot sample X^*b^ = (*X*_1_^*b^, *X*_2_^*b^…, *X*_N_^*b^) (b is one bootstrap [b = 1, 2…, B]) represents a bootstrap sample of size N that has been randomly extracted from **X**, and X^*b^** (0)** is the remaining sample of data **X** that did not appear in X^*b^. AUC (X*^b^, X*^b^** (0)**) represents the AUC of training on patient sample X*^b^ and testing on remaining sample X*^b^** (0)**. This AUC (X*^b^, X*^b^** (0)**) causes a pessimistic bias.

Based on a previous study using 0.632 + bootstrap AUC, we used the top 10 features from the previous selection step^36,37^. We then searched for the optimal model according to the maximum 0.632 + bootstrap AUC using forward feature selection^38,39^. Sensitivity and specificity were also calculated under each condition. The predictive performance of all deep learning models was compared based on the mean AUCs of the 25 models (combinations of five feature selection methods and five machine learning algorithms).

Moreover, a corrected resampled paired t-test^40,41^ was used to evaluate significant differences in the performance of DLR with the highest mean AUC and handcrafted radiomics features and clinical factors. A one-tailed test was used given that we wanted to evaluate whether DLR performed significantly better than handcrafted radiomics features and clinical factors. The calculation of the statistics for the corrected resampled paired t-test requires training and test samples. However, the 0.632 + bootstrap AUC has different training and test sample sizes for each bootstrap. Thus, we used the average of 1000 repetitions for training and test samples: 61 (63.5%) and 35 (36.5%) for GTVp and 50 (63.3%) and 29 (36.7%) for GTVn, respectively. We used the same resampled training and test subset of DLR and handcrafted radiomics features and clinical factors in all 1000 repetitions. The significance level was set at 0.05.

The correlation between the selected 10 features and tumor volume in the initial CT was evaluated using Spearman’s correlation coefficient because useful image features were previously reported to be correlated with tumor volume^42^. We also employed the Gradient Weighted Class Activation Mapping (Grad-CAM) method available in MATLAB to clarify interpretation of deep learning. Specifically, this method can visualize the important regions of interest for the deep learning model.

#### Handcrafted radiomics features

Three types of handcrafted radiomics features, shape, first-order, and texture were extracted from planning CT images of GTVp and GTVn acquired prior to radiotherapy. We used PyRadiomics software^43^ Version 4.10.2 with 3D Slicer to extract the handcrafted radiomics features, and a total of 107 features were extracted from each GTVp and GTVn CT image after resampling to 1 × 1 × 1 mm^3^ (14 shape features, 18 first-order features, and 75 texture features). In turn, texture features were of several types, gray-level co-occurrence matrix (GLCM, n = 24), gray-level run length matrix (GLRLM, n = 16), gray-level dependence matrix (GLDM, n = 14), gray-level size zone matrix (GLSZM, n = 16), and neighborhood gray tone difference matrix (NGTDM, n = 5). The bin width parameter was set to 25 HU. PyRadiomics was chosen for this study because most handcrafted radiomics features extracted are based on the imaging biomarker standardization initiative (IBSI), which provides a benchmark for easy comparison with other studies^44^. However, it appeared that four of the 107 handcrafted radiomics features were not based on IBSI from previous reports^45,46^. All handcrafted radiomics features used in this study are shown in Supplementary Table 4. We used the same methods as described in the Feature selection, Machine learning prediction, and Evaluation sections to select handcrafted radiomics features and predict regression versus nonregression using the 25 models. Model performance was then compared using the mean of 0.632 + bootstrap AUC with 1000 iterations.

### Clinical factors

The following clinical factors were retrospectively collected: age, sex, tumor site, TNM stage, treatment strategy (radiation therapy for purposes of radical, postoperative recurrence), presence of multiple cancers, use of Percutaneous Endoscopic Gastrostomy, implementation of chemotherapy, GTVp volume, GTVn volume, and human papillomavirus (HPV) status (for patients with oropharyngeal cancer). For all patients, the TNM stage was based on the Union for International Cancer Control (UICC) 8th edition. We did not select clinical factors using the multiple segmentation and Spearman’s redundant feature methods described in previous sections because the number was already small (12 features); thus, there was little risk of “curse of dimensionality”^47^. We used five algorithm-based feature selection methods to rank the top 10 clinical factors, and used the same methods as described in the Machine learning prediction and Evaluation sections to predict regression versus nonregression using the 25 models. For evaluation, we also used the average of 0.632 + bootstrap AUC with 1000 iterations.

### Classification accuracy of DLR in different threshold

The median regression rates of GTVp and GTVn were used as thresholds for classification into regression and nonregression groups. However, if different regression rates were used as thresholds for classification, we would expect different AUC results and prediction performance. Therefore, as an additional analysis, we evaluated the predictive performance of the DLR approach using multiple thresholds for GTVp (0.46% [median threshold], 0.8%, 1.2%, 2.0%, 2.4%, 2.8%, and 3.2% per treatment day) and GTVn (1.4% [median threshold], 1.7%, 2.1%, 2.5%, 2.9%, 3.3%, and 4.1% per treatment day). These thresholds were the boundary values that label the two classes of regression and nonregression. However, as the threshold for group inclusion is increased, the number of samples classified into that group becomes smaller and the machine learning prediction may tend to be biased toward the majority class (e.g., nonregression). Therefore, to avoid the minority class being ignored by machine learning, we applied a random under-sampling strategy to balance the classes by down-sampling the majority class to the same size as the minority class^48^. To balance the sample distribution, 100 random under-samplings of the majority class were performed and the final AUC was obtained by averaging 0.632 + bootstrap AUC with 1000 iterations.

## Results

### Total regression and regression rates in GTVp and GTVn cohorts

The median number of treatment days from the initial planning CT to the boost CT scan was 15 (range, 11–20). Mean GTVp was 23.1 cm^3^ on the initial CT images and 20.8 cm^3^ on the boost CT images, while mean GTVn was 33.3 cm^3^ on the initial CT images and 31.9 cm^3^ on the boost CT images. The median relative GTVp regression from the initial CT to the boost CT was 7.17%, and the median GTVn regression over the same period was 20.05%. The median regression rates were 0.46%/treatment day for GTVp and 1.40%/treatment day for GTVn. The median ΔGTVp⁄treatment day in the regression group was 2.02% (30.3% at a median of 15 days) and that in the nonregression group was − 0.20% (− 3% at a median of 15 days) (*P* < 0.0001, Wilcoxon rank sum test). The median ΔGTVn⁄treatment day in the regression group was 3.62% (54.3% at a median of 15 days) and that in the nonregression group was − 0.15% (− 2.25% at a median of 15 days), respectively (*P* < 0.0001, Wilcoxon rank sum test). Thus, both GTVp and GTVn cohorts were stratified into clear regression and nonregression groups during early radiotherapy.

### Regression prediction accuracies for deep learning models

**Figure 3:**
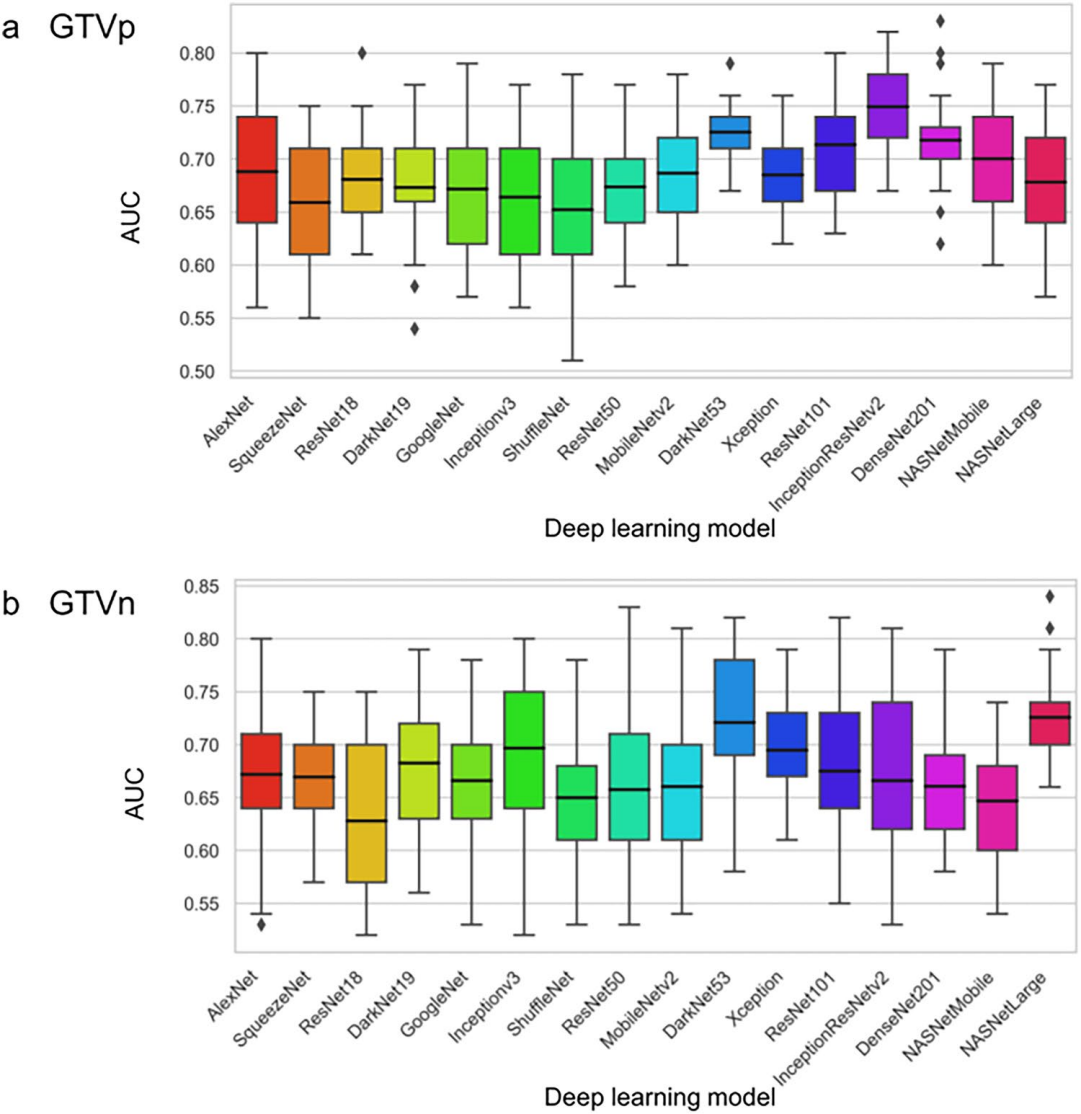
Average performance of 16 CNNs for predicting radiation-induced gross tumor volume (GTV) regression prior to treatment using all combinations of five deep feature selection algorithms and five machine learning algorithms (25 models per CNN). Performance was evaluated by the median area under the receiver operating characteristic (ROC) curve. (**a**) Predictive performance for primary gross tumor volume (GTVp) regression. (**b**) Predictive performance for nodal gross tumor volume (GTVn) regression.

The robust features (ICC > 0.7) selected by each of the 16 pretrained deep learning models from CT images with multiple segmentations are shown in Supplementary Fig. 2. The ICCs of the selected deep features were distributed over a wide range. The AUCs of the 25 models based on each CNN (combinations of five feature selection methods and five machine learning algorithms) for predicting regression of GTVp (Fig. 3a) and GTVn (Fig. 3b) also varied markedly. For the GTVp, highest mean AUC (a measure of average classification accuracy) was achieved using InceptionResNetv2 (followed by DarkNet53 and DenseNet201), while NASNetLarge yielded the highest mean AUC for GTVn regression prediction (followed by DarkNet53 and Inceptionv3). The mean AUCs of the top five models for GTVp classification exceeded 0.7, while two models yielded mean AUCs exceeding 0.7 for GTVn classification. For both GTVp and GTVn classification, the difference in AUC between the worst and best performing model was 0.1. The detailed AUCs for each of the five algorithm-based feature selections and five machine learning algorithms are shown in Supplementary Figs. 3 and 4. The correlation coefficients between the selected 10 features in all deep learning models and tumor volume in the initial CT are shown in Supplementary Tables 5 and 6. As shown, all selected features had very weak correlations with tumor volume.

**Figure 4:**
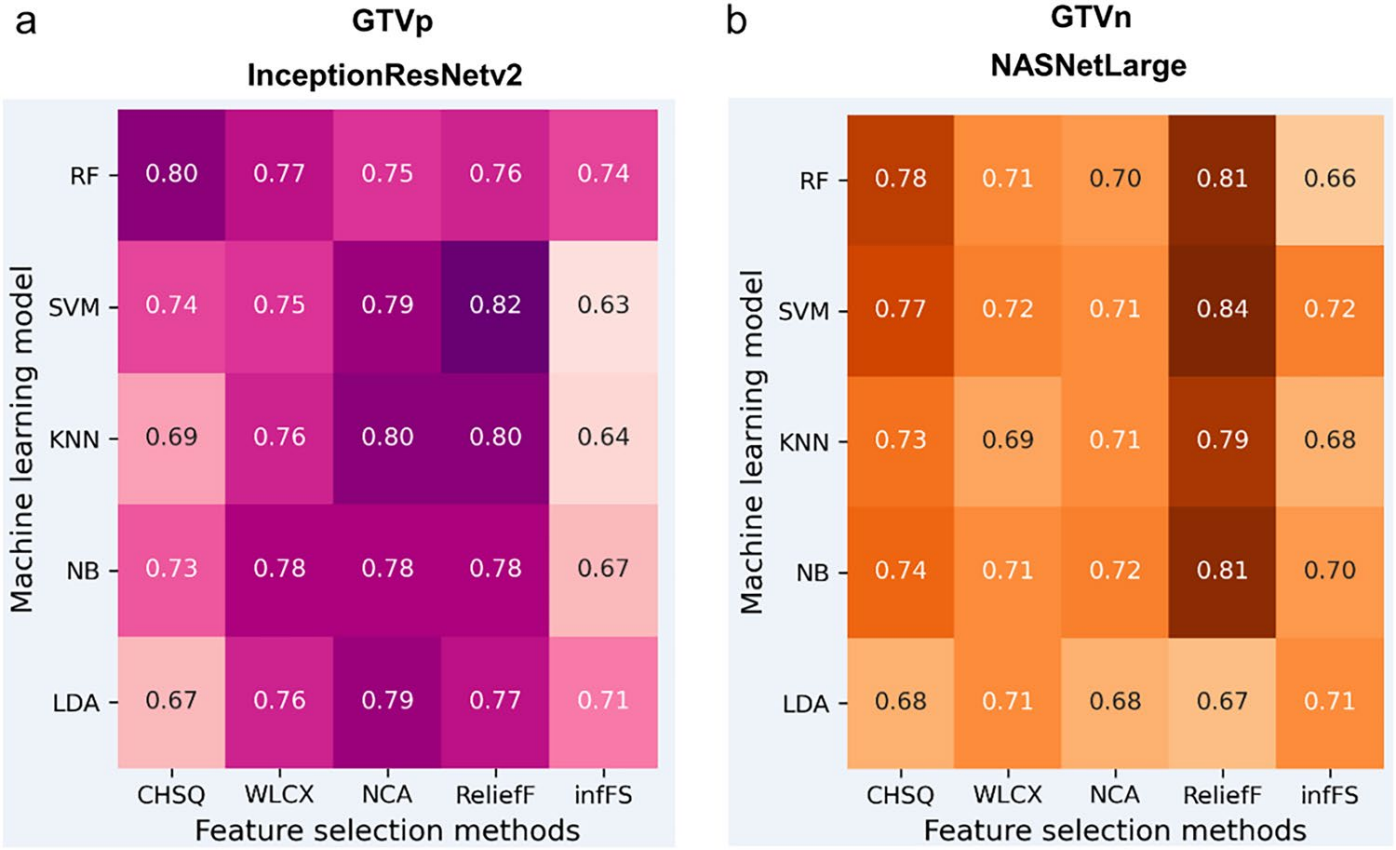
Optimal predictive performance of InceptionResNetv2- and NASNetLarge-based models for predicting GTVp and GTVn regression, respectively. (**a**) Heatmap of the AUCs yielded by 25 InceptionResNetv2-based models (all combinations of five machine learning algorithms in rows and five feature selection algorithms in columns) predicting GTVp regression. (**b**) Corresponding heatmap of AUCs for the 25 NASNetLarge-based models predicting GTVn regression.

Figure 4 presents the individual AUCs for all combinations of the five feature selection algorithms and the five deep learning algorithms using the CNNs yielding highest mean AUCs (InceptionResNetv2 for GTVp and NASNetLarge for GTVn). Notably, AUC values varied widely among combinations of feature selection algorithms and machine learning algorithms, even when using InceptionResNetv2 (Fig. 4a) and NASNetLarge (Fig. 4b) as the CNN.

### Comparisons of prediction accuracy among DLR, handcrafted radiomics feature, and clinical factor models

Table 1 compares the predictive accuracies of our DLR models with the handcrafted radiomics feature-based and clinical factor-based models according to 0.632 + bootstrap AUC, sensitivity, and specificity. The mean AUC of the 25 models (combinations of five feature selection methods and five machine learning algorithms) yielded by InceptionResNetv2 for GTVp regression prediction was larger (mean AUC = 0.75) than the mean AUC yielded by the handcrafted radiomics feature-based models (0.63) or clinical feature-based models (0.64). The predictive accuracy was not substantially improved by the combination of the InceptionResNetv2 plus handcrafted radiomics features (mean AUC = 0.74), clinical factors (0.75), or both (0.75).

**Table 1:** Mean 0.632 + bootstrap areas under the curve (AUCs), sensitivity, and specificity of the deep learning-based radiomics, handcrafted radiomics features, clinical factors, and combined models for predicting primary gross tumor volume (GTVp) regression.

	Mean AUC	Mean sensitivity	Mean specificity
Inceptionresnetv2	0.75 (SD, 0.05)	0.72 (SD, 0.08)	0.66 (SD, 0.08)
Handcrafted radiomics features	0.63 (SD, 0.06)	0.62 (SD, 0.04)	0.60 (SD, 0.05)
Clinical factor	0.64 (SD, 0.04)	0.65 (SD, 0.04)	0.59 (SD, 0.05)
Inceptionresnetv2 + Handcrafted radiomics features	0.74 (SD, 0.06)	0.70 (SD, 0.08)	0.65 (SD, 0.07)
Inceptionresnetv2 + Clinical factor	0.75 (SD, 0.05)	0.72 (SD, 0.07)	0.65 (SD, 0.08)
Inceptionresnetv2 + Handcrafted radiomics features + Clinical factor	0.75 (SD, 0.07)	0.72 (SD, 0.07)	0.65 (SD, 0.08)

*AUC* area under the curve, *SD* standard deviation.

The results of the evaluation of the significant differences in performance between the InceptionResNetv2 for GTVp regression prediction and handcrafted radiomics features and clinical factors are shown in Supplementary Table 7. In the 25 models (5 machine learning models × 5 algorithm-based feature selection), some InceptionResNetv2-based models performed predominantly well, with statistically significant differences. However, no significant differences were observed with the other models.

Table 2 provides the same comparisons for prediction of GTVn regression using NASNetLarge. Again, the mean AUC of the 25 models yielded by NASNetLarge for GTVn regression prediction (mean AUC = 0.73) was larger than that yielded by the mean handcrafted radiomics feature-based model (0.61) or the mean clinical factor-based model (0.67). The prediction was not improved by the combination of the NASNetLarge and handcrafted radiomics features (0.70), clinical factor model (0.71), or both (0.69).

**Table 2:** Mean 0.632 + bootstrap AUCs, sensitivity, and specificity of the deep learning-based radiomics, handcrafted radiomics features, clinical factors, and combined models for predicting nodal gross tumor volume (GTVn) regression.

	Mean AUC	Mean sensitivity	Mean specificity
Nasnetlarge	0.73 (SD, 0.05)	0.70 (SD, 0.06)	0.65 (SD, 0.07)
Handcrafted radiomics features	0.61 (SD, 0.06)	0.63 (SD,0.06)	0.60 (SD, 0.05)
Clinical factor	0.67 (SD, 0.04)	0.65 (SD, 0.04)	0.62 (SD, 0.05)
Nasnetlarge + Handcrafted radiomics features	0.70 (SD, 0.07)	0.69 (SD, 0.07)	0.64 (SD, 0.07)
Nasnetlarge + Clinical factor	0.71 (SD, 0.06)	0.69 (SD, 0.06)	0.63 (SD, 0.08)
Nasnetlarge + Handcrafted radiomics features + Clinical factor	0.69 (SD, 0.07)	0.68 (SD, 0.07)	0.62 (SD, 0.07)

*AUC* area under the curve, *SD* standard deviation.

The results of the evaluation of significant differences in performance between the NASNetLarge for GTVn regression prediction and handcrafted radiomics features and clinical factors are shown in Supplementary Table 8. Almost similar to the GTVp, some NASNetLarge models performed predominantly well in the 25 models (5 machine learning models × 5 algorithm-based feature selection), with some showing statistically significant differences. However, no significant differences were observed with the other models.

### Classification accuracy of DLR models using different ΔGTV thresholds

In this study, prediction was performed by dividing cases into regression and nonregression groups based on median ΔGTV/treatment day as the threshold. However, AUCs (predictive accuracy) may differ if classifications are performed based on other thresholds. Supplementary Tables 9 and 10 show the classification performances of InceptionResNetv2 and NASNetLarge (the CNNs yielding the highest mean AUCs for GTVp and GTVn regression prediction, respectively) using a series of thresholds. For both GTVp and GTVn, there were no significant changes in AUC even when the threshold was increased, and the AUC was above 0.8 at all thresholds. Further, the highest sensitivity was observed using the highest threshold (Supplementary Tables 9 and 10).

### Activation maps of the optimal DLRs

**Figure 5:**
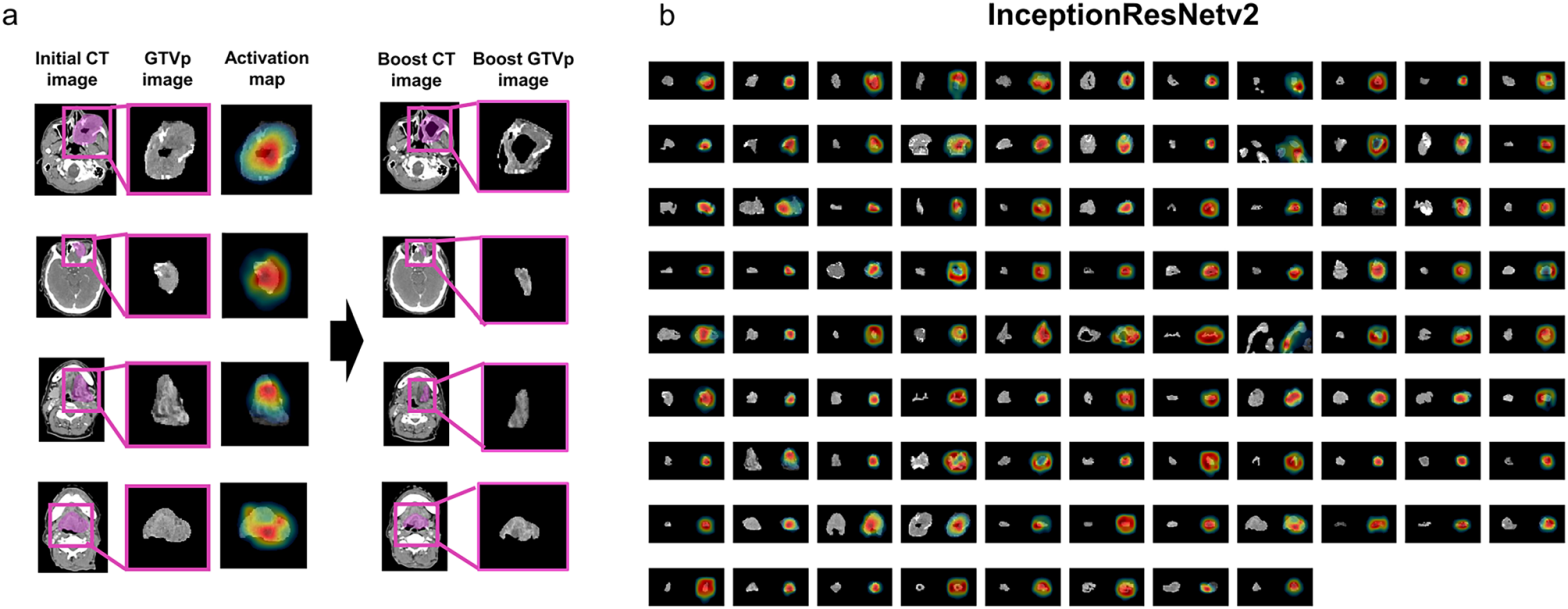
Activation maps of the initial CT images reveal salient features used by InceptionResNetv2-based models for prediction of GTVp regression. (**a**) Activation map of the initial CT image from patients with large GTVp regression using InceptionResNetv2 (the CNN yielding the highest predictive accuracy). The map was visualized using the Gradient Weighted Class Activation Mapping method. The boost CT images are shown to indicate the degree of regression. (**b**) Activation map of the initial GTVp images for all patients yielded by InceptionResNetv2.

Finally, we constructed activation maps of initial CT images using InceptionResNetv2, the CNN yielding highest predictive accuracy for GTVp regression to reveal the most salient image features distinguishing regressing from nonregressing cases (Fig. 5). The activation map of the initial CT images from a patient with particularly large GTVp regression (Fig. 5a) as visualized by the Grad-CAM method revealed that InceptionResNetv2 focuses on the fine characteristics and localized regions of the tumor, such as the tumor interior, tumor edges, and low- and high-density regions (Fig. 5b).

**Figure 6:**
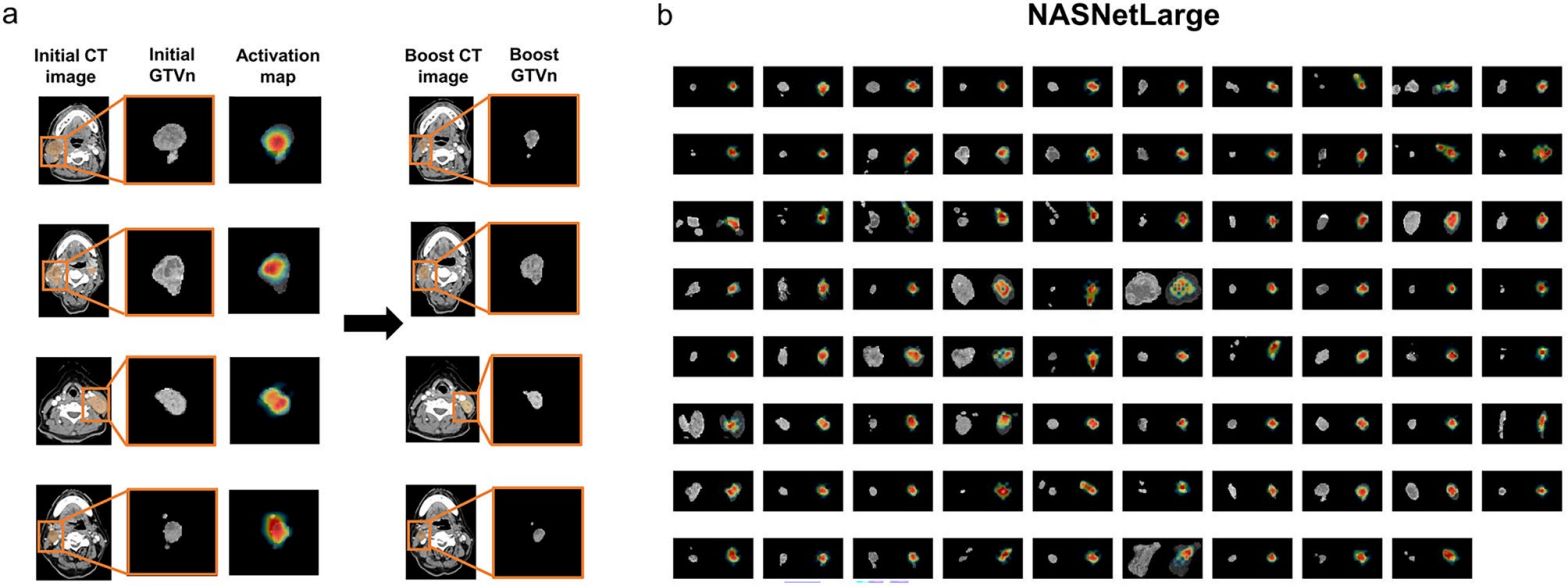
Activation maps of the initial CT images reveal salient features used by NASNetLarge-based models for prediction of GTVn regression. (**a**) Activation map of the initial CT image from a patient with large GTVn regression using NASNetLarge, the CNN yielding the highest prediction accuracy. The boost CT images are shown to illustrate the degree of regression. (**b**) Activation maps for all patients using NASNetLarge.

Similar analyses were conducted for initial CT images of GTVn using NASNetLarge, the CNN yielding highest accuracy for prediction of GTVn regression (Fig. 6). The activation map from a patient with particularly large GTVn regression revealed that like InceptionResNetv2, NASNetLarge focuses on the tumor interior (especially localized areas) rather than the entire tumor (Fig. 6a). The activation maps of the initial GTVn images from all patients revealed that NASNetLarge focuses consistently on the tumor interior (Fig. 6b). In addition, NASNetLarge was able to focus on each GTVn when there were multiple nodal tumors in the image.

## Discussion

To facilitate ART specifically for patients with head and neck cancer who demonstrate early and extensive tumor shrinkage, we developed a DLR approach based solely on pretreatment CT images and demonstrated good potential for predicting early GTVp and GTVn regression.

We compared the predictive efficacies of multiple deep learning models constructed using 16 pretrained CNNs, five feature selection algorithms, and five machine learning algorithms (400 distinct DLR models in total) as well as models based on clinical factors, handcrafted radiomics metrics, and various combinations. The CNNs InceptionResNetv2 and NASNetLarge achieved greater predictive accuracy (reflected by higher mean AUCs in receiver operating characteristic analysis) than other deep learning models, clinical factor-based models, and handcrafted radiomics feature-based models. Further, combining clinical factor- and handcrafted radiomics feature-based models with deep learning models did not substantially improve accuracy (Tables 1 and 2). These findings indicate that deep features extracted from pretrained models may be able to characterize various complex patterns within tumors (tumor heterogeneity) predictive of early radiation-induced regression or radiation resistance.

Surucu et al. developed a decision tree algorithm to predict GTVp and GTVn shrinkage based on clinical factors and demonstrated 88% accuracy^5^. They concluded that factors such as chemotherapy, age, and tumor site are the important predictors of GTVp shrinkage and that factors such as Karnofsky Performance Status, site, and age are the important predictors of GTVn shrinkage. In the present study, the clinical factors ranked highest by CHSQ and yielding the largest AUC for predicting GTVp regression were age, chemotherapy status, and T-stage, and those ranked highest by NCA feature selection and yielding largest AUC for predicting GTVn were tumor site, chemotherapy status, and age, generally consistent with the clinical factors proposed by Surucu et al. Yu et al. also reported that handcrafted radiomics features could predict ART and non-ART groups among patients with nasopharyngeal cancer with high accuracy (AUC = 0.93)^9^. Alves et al. reported that a model combining handcrafted radiomics features and clinical factors predicted ART and non-ART groups with an AUC of 0.84 among patients with head and neck cancer^10^. In both studies, the criteria for ART included factors such as weight loss, lymph node regression, neck tissue loss, and discrepancy in neck contour as well as tumor regression, while the present study focused only on early tumor regression during radiotherapy for predicting ART eligibility. We suggest that future improvements in these DLR models may also allow for the prediction of neck volume shrinkage, shrinkage and positional changes of OARs, and ART eligibility as well as primary and nodal tumor regression.

This DLR strategy also demonstrated higher predictive accuracy than models based on handcrafted radiomics features previously suggested as useful biomarkers for tumor regression (Tables 1 and 2). To the best of our knowledge, the present study is the first to adopt DLR to predict early regression of head and neck tumors during radiotherapy. Deep learning may detect heterogeneity in medical images reflecting genetic and physiological tumor physiological not easily recognized by visual analysis^19^, resulting in greater predictive performance. Another major advantage over handcrafted radiomics features is that deep learning can automatically detect localized regions of the tumor. It is common to analyze the entire tumor when extracting handcrafted radiomics features because there may be no a priori markers to focus attention^45,46,49^, but several studies have reported clinically significant sub-volumes with subtle imaging manifestations, such as hypoxic sub-volumes that are radioresistant^50^ even within a single head and neck tumor^51^. Therefore, visual analyses of total tumor metrics (typical handcrafted radiomics features) may miss important local features that reflect genetic or physiological heterogeneity relevant to therapeutic response and prognosis. Conversely, deep learning automatically detects specific image patterns within the tumor learned from natural images (Figs. 5 and 6). In other words, deep learning may automatically detect subregions related to radiation sensitivity or resistance, thus distinguishing patients with early regression or nonregression.

The mean AUCs among deep learning models differed by up to 0.1, with InceptionResNetv2 yielding the highest mean AUC for prediction of GTVp regression (Fig. 3a). InceptionResNetv2 is a hybrid CNN (164 layers) that combines the Inception and ResNet modules. The Inception module extracts features from images at various resolutions, and the ResNet module (residual connection) extracts complicated features from the deep layers of the CNN. Pretrained InceptionResNetv2-based models have demonstrated excellent prediction accuracy using x-ray^52^ and ultrasound images^53^ as inputs, and this study extends this predictive potential to CT images.

NASNetLarge achieved the highest mean AUC among all models for predicting GTVn regression (Fig. 3b). NASNetLarge automatically learns the model architecture and designs the optimal structure for ImageNet classification^54^. It has the deepest layered structure and the largest number of parameters among the models used in this study, and because of its deep layers and large number of parameters, we could extract many phenotypes. Kornblith reported that NASNetLarge achieves top-of-class accuracy for ImageNet image classification^55^. When there were multiple GTVn in the tumor images, NASNetLarge was able to focus on every tumor (Fig. 6b).

With any network, there is a risk that the results using a single algorithm may lack classification accuracy on other datasets. Therefore, it is important to provide benchmarks using various networks, feature selection algorithms, and machine learning algorithms to identify the best approach for each task or modality. In future studies, it will be necessary to standardize DLR approaches. The results obtained from deep learning models may also depend on image size, window level settings, and machine learning and feature selection algorithms, so further analyses, such as comparisons using larger datasets or benchmark validation with independent external datasets, is necessary to determine the optimal model for predicting tumor regression from planning CT images in a wide variety of clinical situations. 

In this study, we used thresholds of 0.46%/treatment day and 1.4%/treatment day for distinguishing GTVp and GTVn regression from nonregression, respectively. The median GTV regression rate from the initial CT scan to the boost CT scan was 7.17% for GTVp and 20.05% for GTVn. Other studies have reported GTV regression rates ranging from 3 to 16% for up to 10 treatment days and 7–48% for up to 20 treatment days^4^, so the regression rates observed in our study cohorts were relatively slow. Schwartz et al. reported that ART reduced mean dose to the contralateral parotid gland by 0.6 Gy (2.9%) and mean dose to the ipsilateral parotid gland by 1.3 Gy (3.8%) compared to image-guided radiation therapy alone in patients with median CTV volume reduction of only 5%^56^. In addition, Bhide et al. reported a significantly lower mean minimum dose to the PTV and high dose heterogeneity among patients with a modest 3.2% reduction in macroscopic CTV over two weeks^2^. Therefore, prediction of regression by our DLR approach may improve the minimum dose and heterogeneity of PTV and parotid dose for patients receiving ART, even if regression rate is low. In addition, when the classification threshold was higher than the median, precision was maintained and sensitivity was slightly improved (Supplementary Tables 9 and 10). Therefore, we believe that DLR has great potential for predicting early radiation-induced tumor regression prior to radiotherapy. DLR may facilitate truly personalized adaptive radiotherapy for patients showing early GTVp and GTVn regression.

This study is subject to several limitations. First, the number of patients (96 for GTVp and 79 for GTVn monitoring) was insufficient to train the models and evaluate the accuracy using a subset of cases as an external test dataset. To select deep features associated with tumor regression, from a large number of deep features in each deep learning model, we used the entire dataset for redundant feature selection and algorithm-based feature selection steps. Therefore, it should be noted that the results of this study are an internal validation and the performance obtained may have an optimistic bias. It will be necessary to train feature selection and models on a larger amount of training data and then validate them using an independent external validation dataset to ensure that these results are generalizable. Further, the results were generated from a single institution. To broaden the applicability of these results, validation at multiple institutions is required. It is also difficult to interpret the pathophysiological and clinical significance of deep features. While we generated activation maps to establish the reliability of the CNN models, we have not established associations between specific patterns and the underlying processes determining the rate of radiation-induced tumor regression. Understanding the clinical implications of these deep features is necessary to incorporate DLR into routine clinical practice. Therefore, as a next step, it is important to identify associations between deep features — such as regions of tumor heterogeneity appearing on CT images (e.g., intensity patterns) — and regional genetic or physiological variation (i.e., the pathogenic processes reflected by these deep features). We were only able to extract deep features from one axial slice because most CNN model layers pretrained on natural image datasets were constructed with 2D inputs. Therefore, if information about tumor regression was included for other axial slices, important information may be missed. In the future, the development of existing models that use 3D images as input will solve this problem.

In conclusion, we developed and evaluated a deep learning radiomics approach to predict early regression of GTVp and GTVn during radiotherapy based on planning CT images. Although there are some limitations, our results suggest that the proposed method is effective for identifying patients requiring ART.

## Supplementary Information

Supplementary Information.
