# Supplementary material for: A deep learning-based radiomics approach to predict head and neck tumor regression for adaptive radiotherapy
Source: Sci Rep. 2022 May 27;12:8899. doi: 10.1038/s41598-022-12170-z (PMC9142601; doi:10.1038/s41598-022-12170-z)
Supplement: Supplementary file 1 — Supplementary Information. [file 41598_2022_12170_MOESM1_ESM.pdf]

Supplementary information

A deep learning-based radiomics approach to predict head and neck tumor regression for adaptive radiotherapy

Shohei Tanaka, M.S.,[1] Noriyuki Kadoya*, Ph.D.,[1] Yuto Sugai, B.S.,[1]
Mariko Umeda, B.S.,[1] Miyu Ishizawa, B.S.,[2] Yoshiyuki Katsuta, Ph.D.,[1] Kengo Ito, M.S.,[1] Ken Takeda, M.D., Ph.D.,[2] Keiichi Jingu, M.D., Ph.D.,[1]

(1) Department of Radiation Oncology, Tohoku University Graduate School of Medicine, Sendai, Japan
(2) Department of Radiological Technology, School of Health Sciences, Faculty of Medicine, Tohoku University, Sendai, Japan

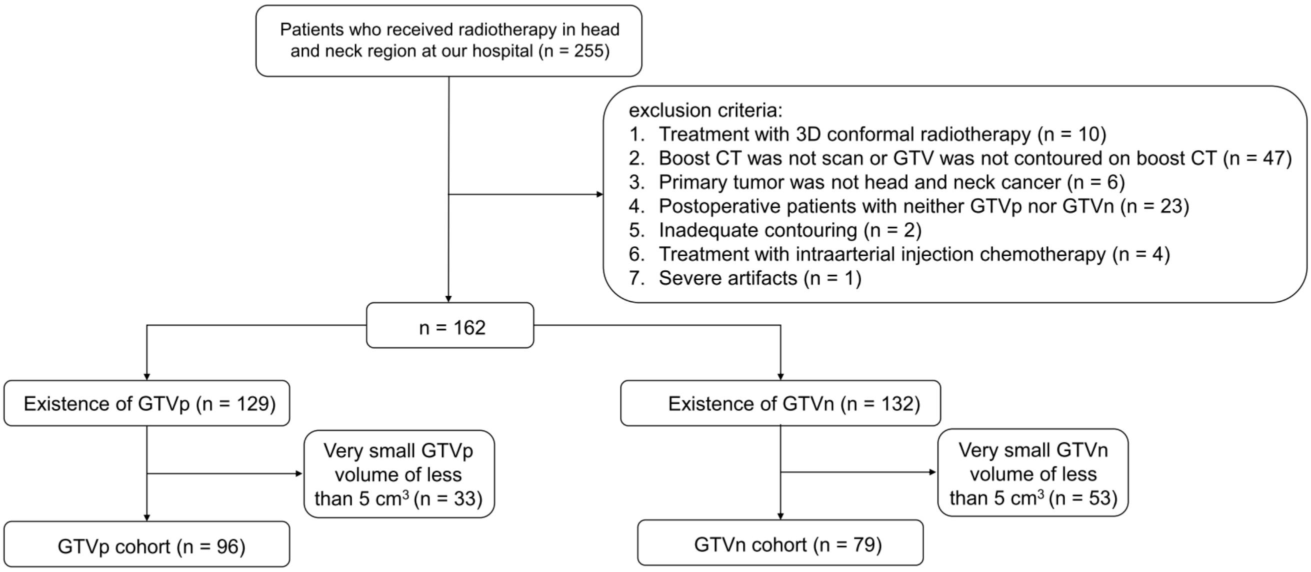

Supplementary Figure S1: **Inclusion and exclusion criteria for study enrolment.** GTVp: primary gross tumor volume; GTVn: nodal gross tumor volume

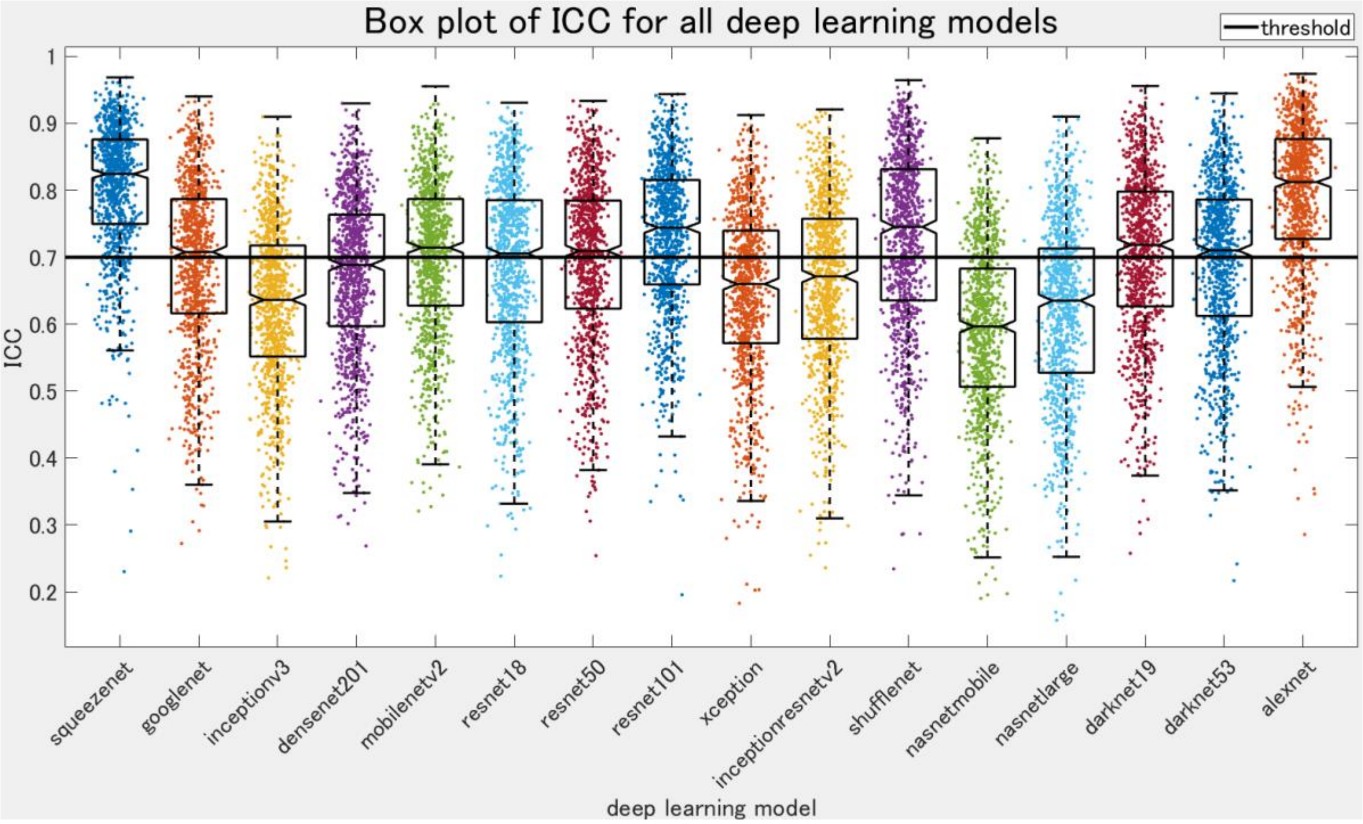

Supplementary Figure S2: **Intraclass correlation coefficients (ICCs) for features extracted by the 16 convolution neural network (CNN) deep learning models**. The x-axis identifies the deep learning model and the y-axis is the ICC. Deep features were extracted from a multiple segmentation dataset [1] by each deep learning model, and evaluated for robustness. Deep features with ICC > 0.7 were regarded as robust.

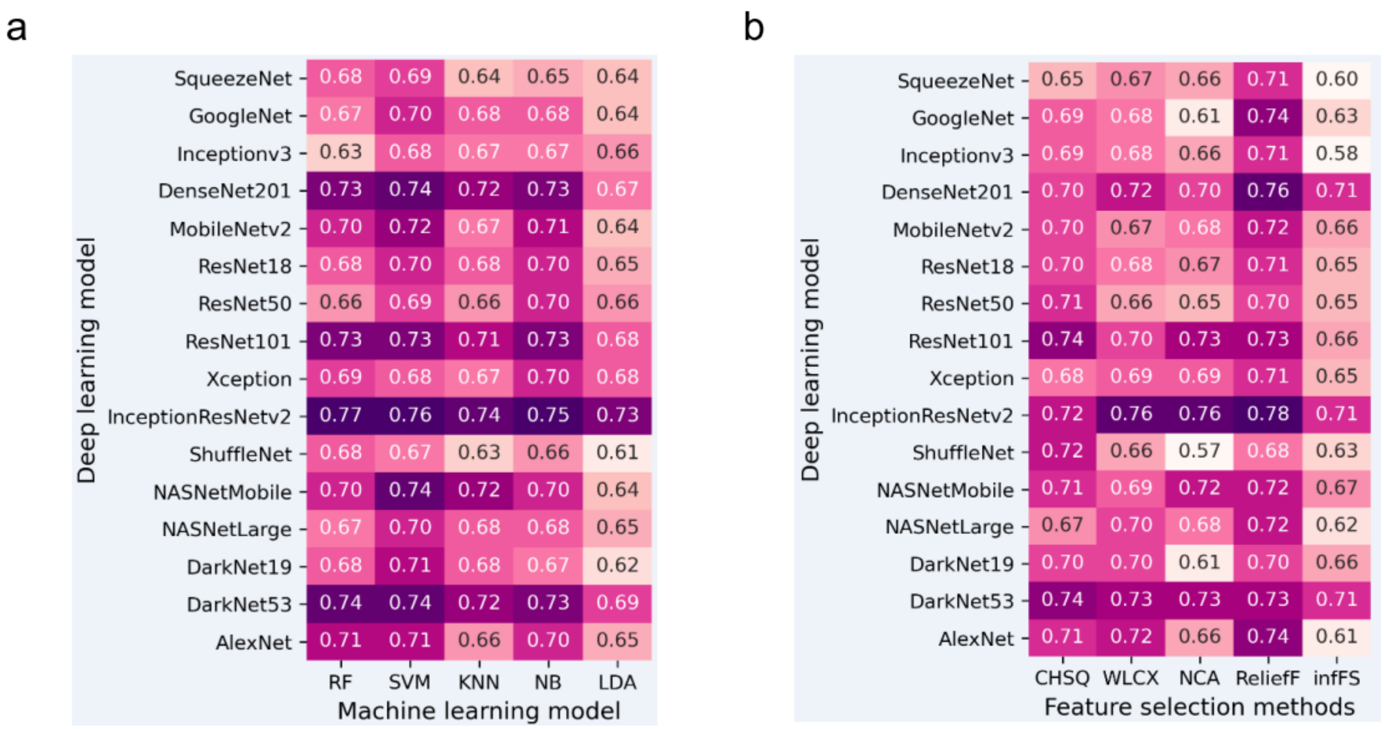

Supplementary Figure S3: **Areas under the curve (AUCs) for GTVp regression prediction yielded by all 16 deep learning models using each of the five machine learning algorithms and the five feature selection algorithms**. (a) Heatmap of AUCs yielded by the indicated deep learning model (row) using the indicated machine learning algorithm (column) and all five feature selection algorithms (i.e., each AUC is the average of five feature selection algorithms). (b) Heatmap of mean AUCs yielded by each deep learning model (row) and the indicated feature selection algorithm (column) for all five machine learning algorithms (i.e., each AUC is the average of five machine learning algorithms).

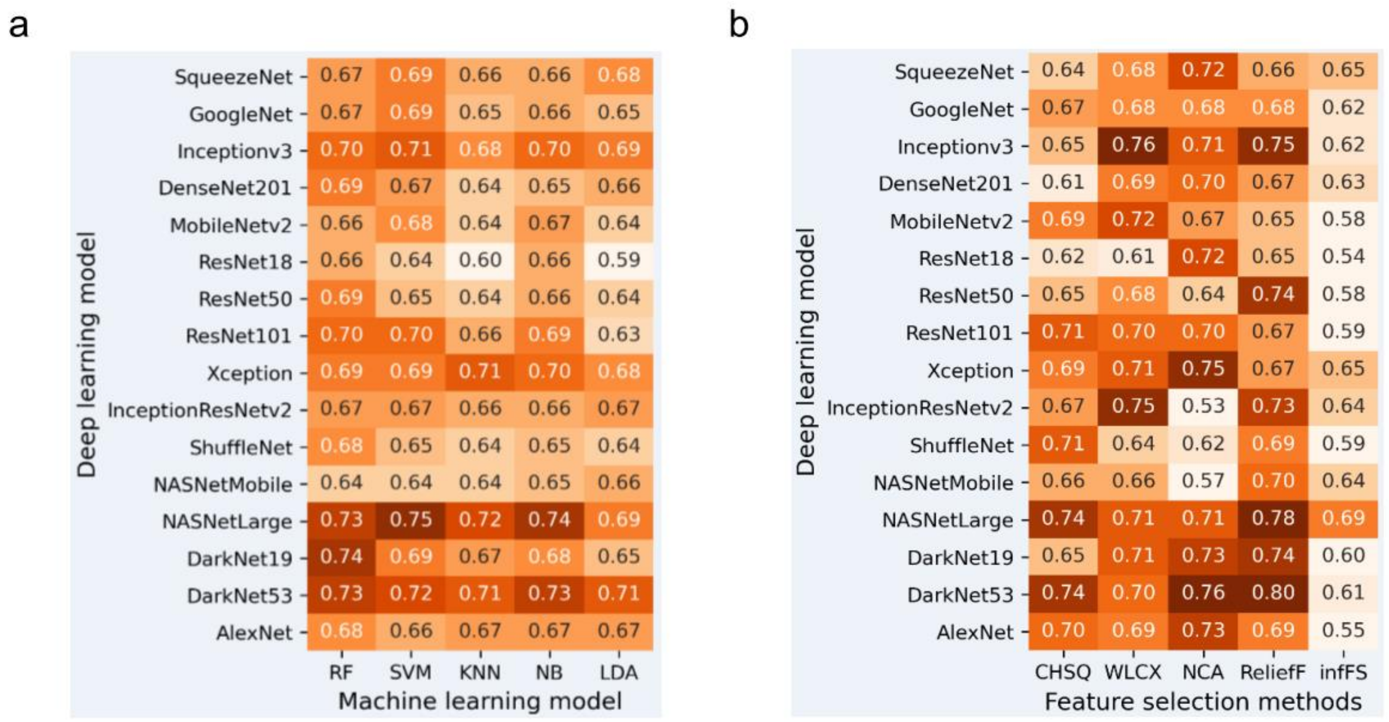

Supplementary Figure S4: **Areas under the curve (AUCs) for GTVn prediction yielded by all 16 deep learning models using each of the five machine learning algorithms and the five feature selection algorithms**. (a) Heatmap of the AUCs yielded by the indicated deep learning model (row) using the indicated machine learning algorithm (column) and all five feature selection methods (i.e., each AUC is the average of 5 feature selection algorithm). (b) Heatmap of the AUCs yielded by the indicated deep learning model (row) using the indicated feature selection algorithm (column) and all five machine learning algorithms (i.e., each AUC is the average of 5 machine learning algorithm).

Supplementary Table S1: **Characteristics of the GTVp cohort**

Characteristic: GTVp		Overall n = 96
Sex	Male	81(84.4%)
	Female	15(15.6%)
Age (years: mean ± SD)		67.1 ± 10.9
Site	nasopharynx	13(13.5%)
	oropharynx	31(32.3%)
	hypopharynx	24(25.0%)
	oral cavity	18(18.8%)
	larynx	7(7.3%)
	paranasal sinus	3(3.1%)
Oropharynx carcinoma HPV16	Positive	10(10.4%)
	Negative	21(21.9%)
	–	65(67.7%)
Treatment	Radical radiotherapy	80(83.3%)
	Postoperative recurrence radiotherapy	16(16.7%)
T stage	T1	11(11.5%)
	T2	23(24.0%)
	T3	20(20.8%)
	T4	6(6.3%)
	T4a	13(13.5%)
	T4b	8(8.3%)
	–	15(15.6%)
N stage	N0	12(12.5%)
	N1	22(22.9%)
	N2	11(11.5%)
	N2a	2(2.1%)
	N2b	15(15.6%)
	N2c	6(6.3%)
	N3	2(2.1%)
	N3b	11(11.5%)
	–	15(15.6%)
M stage	M0	94(97.9%)
	M1	2(2.1%)
Chemotherapy	Done	67(69.8%)
	No	29(30.2%)
Multiple primary cancer	Yes	27(28.1%)
	No	69(71.9%)
PEG (Percutaneous Endoscopic Gastrostomy)	Yes	63(65.6%)
	No	33(34.4%)
GTVp volume (cm^3: mean ± SD)		23.1 ± 22.3

Supplementary Table S2: **Characteristics of the GTVn cohort**

Characteristic: GTVn		Overall n = 79
Sex	Male	71(89.9%)
	Female	8(10.1%)
Age (years: mean ± SD)		67.4 ± 9.98
Site	nasopharynx	13(16.5%)
	oropharynx	21(26.6%)
	hypopharynx	22(27.8%)
	oral cavity	15(19.0%)
	larynx	8(10.1%)
	paranasal sinus	0
Oropharynx carcinoma HPV16	Positive	17(21.5%)
	Negative	3(3.8%)
	-	59(74.7%)
Treatment	Radical radiotherapy	65(82.3%)
	Postoperative recurrence radiotherapy	14(17.7%)
13T stage	T0	1(1.3%)
	T1	16(20.3%)
	T1b	2(2.5%)
	T2	16(20.3%)
	T3	14(17.7%)
	T4	4(5.1%)
	T4a	9(11.4%)
	T4b	3(3.8%)
	-	14(17.7%)
N stage	N0	1(1.3%)
	N1	16(20.3%)
	N2	7(8.9%)
	N2a	2(2.5%)
	N2b	14(17.7%)
	N2c	7(8.9%)
	N3	6(7.6%)
	N3b	12(15.2%)
	-	14(17.7%)
M stage	M0	77(97.5%)
	M1	2(2.5%)
Chemotherapy	Done	61(77.2%)
	No	18(22.8%)
Multiple primary cancer	Yes	17(21.5%)
	No	62(78.5%)
PEG (Percutaneous Endoscopic Gastrostomy)	Yes	55(69.6%)
	No	24(30.4%)
GTVn volume (cm^3: mean ± SD)		33.3 ± 53.8

Supplementary Table S3: **Hyperparameters of the machine learning models.**

Machine learning model	Description
Random Forest (RF)	The “fitcensemble” function was used in MATLAB. The weak learner used in the ensemble was the template tree. “NumLearningCycle” was set to 100.
Support Vector Machine (SVM)	The “fitcsvm” function was used in MATLAB. The kernel function was the rbf kernel and the kernel scale was Auto.
K-Nearest Neighborhood (KNN)	The “fitcknn” function was used in MATLAB. “NumNeighbors” was set to 10.
Naïve Bayes (NB)	The “fitcnb” function was used in MATLAB. “NumNeighbors” was set to 10. The normal (Gaussian) distribution was used for the data distribution to model the data.
Linear Discriminant Analysis (LDA)	The “fitcdiscr” function was used in MATLAB.

Supplementary Table S4: **All handcrafted radiomics features used in this study (n = 107).**

Category	Feature Name
Shape	Voxel Volume
Shape	Maximum 3D Diameter
Shape	Mesh Volume
Shape	Major Axis Length
Shape	Sphericity
Shape	Least Axis Length
Shape	Elongation
Shape	Surface Volume Ratio
Shape	Maximum 2D Diameter Slice
Shape	Flatness
Shape	Surface Area
Shape	Minor Axis Length
Shape	Maximum 2D Diameter Column
Shape	Maximum 2D Diameter Row
Intensity	Interquartile Range
Intensity	Skewness
Intensity	Uniformity
Intensity	Median
Intensity	Energy
Intensity	Robust Mean Absolute Deviation
Intensity	Mean Absolute Deviation
Intensity	Total Energy
Intensity	Maximum
Intensity	Root Mean Squared
Intensity	90th Percentile
Intensity	Minimum
Intensity	Entropy
Intensity	Range
Intensity	Variance
Intensity	10th Percentile
Intensity	Kurtosis
Intensity	Mean
Texture (GLCM)	Joint Average
Texture (GLCM)	Sum Average
Texture (GLCM)	Joint Entropy
Texture (GLCM)	Cluster Shade
Texture (GLCM)	Maximum Probability

Texture (GLCM)	Inverse Difference Moment Normalized
Texture (GLCM)	Joint Energy
Texture (GLCM)	Contrast
Texture (GLCM)	Difference Entropy
Texture (GLCM)	Inverse Variance
Texture (GLCM)	Difference Variance
Texture (GLCM)	Inverse Difference Normalized
Texture (GLCM)	Inverse Difference Moment
Texture (GLCM)	Correlation
Texture (GLCM)	Autocorrelation
Texture (GLCM)	Sum Entropy
Texture (GLCM)	Maximal Correlation Coefficient
Texture (GLCM)	Sum Squares
Texture (GLCM)	Cluster Prominence
Texture (GLCM)	Informational Measure of Correlation 1
Texture (GLCM)	Informational Measure of Correlation 2
Texture (GLCM)	Difference Average
Texture (GLCM)	Inverse Difference
Texture (GLCM)	Cluster Tendency
Texture (GLRLM)	Short Run Low Gray Level Emphasis
Texture (GLRLM)	Gray Level Variance
Texture (GLRLM)	Low Gray Level Run Emphasis
Texture (GLRLM)	Gray Level Nonuniformity Normalized
Texture (GLRLM)	Run Variance
Texture (GLRLM)	Gray Level Nonuniformity
Texture (GLRLM)	Long Run Emphasis
Texture (GLRLM)	Short Run High Gray Level Emphasis
Texture (GLRLM)	Run Length Nonuniformity
Texture (GLRLM)	Short Run Emphasis
Texture (GLRLM)	Long Run High Gray Level Emphasis
Texture (GLRLM)	Run Percentage
Texture (GLRLM)	Long Run Low Gray Level Emphasis
Texture (GLRLM)	Run Entropy
Texture (GLRLM)	High Gray Level Run Emphasis
Texture (GLRLM)	Run Length Nonuniformity Normalized
Texture (GLSZM)	Gray Level Variance
Texture (GLSZM)	Zone Variance
Texture (GLSZM)	Gray Level Nonuniformity Normalized
Texture (GLSZM)	Size Zone Nonuniformity Normalized

Texture (GLSZM)	Size Zone Nonuniformity
Texture (GLSZM)	Gray Level Nonuniformity
Texture (GLSZM)	Large Area Emphasis
Texture (GLSZM)	Small Area High Gray Level Emphasis
Texture (GLSZM)	Zone Percentage
Texture (GLSZM)	Large Area Low Gray Level Emphasis
Texture (GLSZM)	Large Area High Gray Level Emphasis
Texture (GLSZM)	High Gray Level Zone Emphasis
Texture (GLSZM)	Small Area Emphasis
Texture (GLSZM)	Low Gray Level Zone Emphasis
Texture (GLSZM)	Zone Entropy
Texture (GLSZM)	Small Area Low Gray Level Emphasis
Texture (GLDM)	Gray Level Variance
Texture (GLDM)	High Gray Level Emphasis
Texture (GLDM)	Dependence Entropy
Texture (GLDM)	Dependence Nonuniformity
Texture (GLDM)	Gray Level Nonuniformity
Texture (GLDM)	Small Dependence Emphasis
Texture (GLDM)	Small Dependence High Gray Level Emphasis
Texture (GLDM)	Dependence Nonuniformity Normalized
Texture (GLDM)	Large Dependence Emphasis
Texture (GLDM)	Large Dependence Low Gray Level Emphasis
Texture (GLDM)	Dependence Variance
Texture (GLDM)	Large Dependence High Gray Level Emphasis
Texture (GLDM)	Small Dependence Low Gray Level Emphasis
Texture (GLDM)	Low Gray Level Emphasis
Texture (NGTDM)	Coarseness
Texture (NGTDM)	Complexity
Texture (NGTDM)	Strength
Texture (NGTDM)	Contrast
Texture (NGTDM)	Busyness

GLCM: Gray Level Co-occurrence Matrix; GLRLM: Gray Level Run Length Matrix; GLSZM: Gray Level Size Zone Matrix; GLDM: Gray Level Dependence Matrix; NGTDM: Neighboring Gray Tone Difference Matrix

Supplementary Table S5: **Correlation between selected deep features and the initial GTVp volume in all 16 deep learning models using five feature selection methods.**

	CHSQ	WLCX	NCA	ReliefF	infFS
SqueezeNet	0.06<br>(−F0.18 to 0.15)	−0.01<br>(−0.14 to 0.12)	−0.04<br>(−0.18 to 0.28)	0.11<br>(0.02 to 0.15)	0.07<br>(−0.09 to 0.16)
GoogleNet	−0.02<br>(−0.2 to 0.15)	0.02<br>(−0.16 to 0.13)	0.07<br>(−0.08 to 0.15)	0.02<br>(−0.17 to 0.12)	0.05<br>(−0.12 to 0.20)
Inceptionv3	0.00<br>(−0.22 to 0.13)	−0.02<br>(−0.16 to 0.28)	−0.03<br>(−0.16 to 0.28)	0.01<br>(−0.15 to 0.28)	0.00<br>(−0.22 to 0.19)
DenseNet201	0.05<br>(−0.15 to 0.15)	−0.02<br>(−0.14 to 0.28)	−0.03<br>(−0.22 to 0.15)	0.00<br>(−0.22 to 0.28)	0.02<br>(−0.08 to 0.10)
MobileNetv2	0.02<br>(−0.22 to 0.25)	0.03<br>(−0.24 to 0.22)	−0.09<br>(−0.2 to 0.10)	0.02<br>(−0.2 to 0.14)	−0.06<br>(−0.15 to 0.15)
ResNet18	−0.03<br>(−0.11 to 0.21)	0.00<br>(−0.17 to 0.22)	0.01<br>(−0.16 to 0.21)	−0.01<br>(−0.17 to 0.10)	0.02<br>(−0.21 to 0.20)
ResNet50	0.02<br>(−0.26 to 0.16)	−0.02<br>(−0.15 to 0.17)	−0.03<br>(−0.15 to 0.17)	0.02<br>(−0.15 to 0.17)	0.02<br>(−0.18 to 0.18)
ResNet101	−0.01<br>(−0.19 to 0.06)	0.00<br>(−0.13 to 0.12)	−0.06<br>(−0.12 to 0.09)	0.01<br>(−0.10 to 0.19)	0.02<br>(−0.18 to 0.26)
Xception	0.09<br>(−0.18 to 0.16)	−0.02<br>(−0.18 to 0.22)	0.03<br>(−0.18 to 0.22)	−0.04<br>(−0.18 to 0.17)	−0.06<br>(−0.12 to 0.17)
InceptionResNetv2	0.00<br>(−0.14 to 0.08)	−0.04<br>(−0.09 to 0.14)	−0.04<br>(−0.09 to 0.15)	0.03<br>(−0.08 to 0.13)	0.00<br>(−0.09 to 0.05)
ShuffleNet	−0.03<br>(−0.23 to 0.11)	0.03<br>(−0.2 to 0.14)	0.00<br>(−0.14 to 0.15)	−0.02<br>(−0.1 to 0.13)	−0.01<br>(−0.27 to 0.05)
NASNetMobile	−0.06<br>(−0.15 to 0.17)	0.03<br>(−0.12 to 0.12)	−0.04<br>(−0.1 to 0.17)	−0.06<br>(−0.12 to 0.12)	−0.02<br>(−0.14 to 0.09)
NASNetLarge	0.01<br>(−0.1 to 0.22)	−0.04<br>(−0.1 to 0.17)	0.03<br>(−0.09 to 0.17)	−0.01<br>(−0.08 to 0.08)	0.03<br>(−0.09 to 0.13)
DarkNet19	0.01<br>(−0.12 to 0.09)	−0.01<br>(−0.1 to 0.13)	0.01<br>(−0.16 to 0.20)	−0.07<br>(−0.14 to 0.13)	−0.02<br>(−0.22 to 0.10)
DarkNet53	−0.02<br>(−0.17 to 0.18)	−0.02<br>(−0.17 to 0.26)	0.01<br>(−0.15 to 0.26)	0.02<br>(−0.17 to 0.24)	−0.05<br>(−0.18 to 0.10)
AlexNet	−0.07<br>(−0.14 to 0.15)	−0.04<br>(−0.08 to 0.09)	−0.03<br>(−0.20 to 0.27)	−0.09<br>(−0.26 to 0.08)	0.01<br>(−0.15 to 0.18)

CHSQ: Chi square score; WLCX: Wilcoxon; NCA: Neighborhood Component Analysis; infFS: Infinite Feature Selection.

The median correlations of the 10 selected deep features are shown. The corresponding minimum and maximum values are given in parentheses below the median. All selected deep features had very weak correlations with the primary gross tumor volume (GTVp) volume.

Supplementary Table S6: **Correlations between selected deep features and the initial GTVn volume in all 16 deep learning models using five feature selection methods.**

	CHSQ	WLCX	NCA	ReliefF	infFS
SqueezeNet	0.00 (−0.14 to 0.11)	0.04 (−0.13 to 0.13)	0.01 (−0.18 to 0.18)	0.01 (−0.13 to 0.13)	0.09 (−0.17 to 0.22)
GoogleNet	−0.02 (−0.16 to 0.18)	0.05 (−0.06 to 0.20)	0.02 (−0.16 to 0.14)	0.02 (−0.23 to 0.26)	0.04 (−0.15 to 0.16)
Inceptionv3	−0.05 (−0.21 to 0.09)	−0.05 (−0.09 to 0.20)	−0.03 (−0.13 to 0.09)	−0.05 (−0.14 to 0.05)	−0.02 (−0.10 to 0.12)
DenseNet201	0.00 (−0.09 to 0.15)	−0.04 (−0.10 to 0.08)	−0.02 (−0.09 to 0.33)	0.00 (−0.06 to 0.14)	0.08 (−0.05 to 0.16)
MobileNetv2	−0.06 (−0.16 to 0.19)	−0.01 (−0.18 to 0.17)	−0.03 (−0.17 to 0.17)	−0.02 (−0.17 to 0.12)	−0.08 (−0.17 to 0.17)
ResNet18	0.05 (−0.13 to 0.19)	−0.03 (−0.18 to 0.07)	−0.03 (−0.17 to 0.10)	0.03 (−0.17 to 0.19)	0.01 (−0.17 to 0.17)
ResNet50	−0.01 (−0.14 to 0.10)	−0.05 (−0.19 to 0.06)	0.01 (−0.08 to 0.16)	0.00 (−0.14 to 0.09)	−0.02 (−0.18 to 0.16)
ResNet101	0.00 (−0.13 to 0.12)	−0.06 (−0.13 to 0.08)	0.00 (−0.13 to 0.14)	0.01 (−0.09 to 0.14)	0.02 (−0.04 to 0.17)
Xception	0.01 (−0.12 to 0.15)	0.00 (−0.12 to 0.15)	−0.01 (−0.12 to 0.12)	−0.07 (−0.12 to 0.15)	0.02 (−0.18 to 0.15)
InceptionResNetv2	−0.03 (−0.22 to 0.15)	−0.09 (−0.33 to 0.14)	−0.06 (−0.27 to 0.17)	−0.04 (−0.28 to 0.26)	0.01 (−0.08 to 0.15)
ShuffleNet	−0.06 (−0.18 to 0.12)	0.01 (−0.11 to 0.16)	0.01 (−0.11 to 0.16)	0.00 (−0.19 to 0.16)	0.03 (−0.17 to 0.22)
NASNetMobile	−0.01 (−0.28 to 0.09)	0.02 (−0.28 to 0.15)	−0.03 (−0.13 to 0.15)	0.05 (−0.14 to 0.15)	0.01 (−0.09 to 0.15)
NASNetLarge	−0.04 (−0.16 to 0.09)	−0.01 (−0.14 to 0.06)	−0.02 (−0.14 to 0.08)	−0.04 (−0.14 to 0.09)	0.02 (−0.09 to 0.12)
DarkNet19	0.01 (−0.23 to 0.22)	0.00 (−0.15 to 0.27)	0.00 (−0.14 to 0.12)	0.03 (−0.24 to 0.27)	−0.01 (−0.15 to 0.13)
DarkNet53	−0.05 (−0.20 to 0.03)	0.01 (−0.23 to 0.29)	0.09 (−0.30 to 0.25)	−0.01 (−0.20 to 0.29)	0.09 (−0.11 to 0.21)
AlexNet	0.00 (−0.10 to 0.14)	0.03 (−0.20 to 0.17)	0.06 (−0.06 to 0.17)	0.01 (−0.09 to 0.17)	0.01 (−0.15 to 0.17)

CHSQ: Chi square score; WLCX: Wilcoxon; NCA: Neighborhood Component Analysis; infFS: Infinite Feature Selection.

The median correlations of 10 selected deep features are shown. The corresponding minimum and maximum values are given in parentheses below the median. All selected deep features had very weak correlations with the nodal gross tumor volume (GTVn) volume.

Supplementary Table S7: **Significant differences in the performance (0.632+ bootstrap AUC of 1000 repetitions) between the InceptionResNetv2 and handcrafted radiomics features and clinical factors for GTVp regression prediction.**

Features	Machine learning	Feature selection	p value
InceptionResNetv2 vs. Handcrafted radiomics features	RF	CHSQ	0.021
	RF	WLCX	0.008
	RF	NCA	0.359
	RF	ReliefF	0.112
	RF	infFS	0.147
	KNN	CHSQ	0.183
	KNN	WLCX	< 0.001
	KNN	NCA	0.141
	KNN	ReliefF	0.014
	KNN	infFS	0.003
	SVM	CHSQ	0.102
	SVM	WLCX	0.039
	SVM	NCA	0.085
	SVM	ReliefF	0.161
	SVM	infFS	0.057
	NB	CHSQ	0.195
	NB	WLCX	< 0.001
	NB	NCA	0.118
	NB	ReliefF	0.046
	NB	infFS	0.018
	LDA	CHSQ	0.427
	LDA	WLCX	0.001
	LDA	NCA	0.186
	LDA	ReliefF	0.080
	LDA	infFS	0.235
InceptionResNetv2 vs. Clinical factors	RF	CHSQ	0.063
	RF	WLCX	0.081
	RF	NCA	0.124
	RF	ReliefF	0.112
	RF	infFS	0.197
	KNN	CHSQ	0.187
	KNN	WLCX	0.014
	KNN	NCA	0.031
	KNN	ReliefF	0.008

KNN	infFS	0.244
SVM	CHSQ	0.093
SVM	WLCX	0.032
SVM	NCA	0.009
SVM	ReliefF	0.073
SVM	infFS	0.190
NB	CHSQ	0.194
NB	WLCX	0.037
NB	NCA	0.100
NB	ReliefF	0.070
NB	infFS	0.218
LDA	CHSQ	0.485
LDA	WLCX	0.046
LDA	NCA	0.077
LDA	ReliefF	0.047
LDA	infFS	0.107

RF: Random Forest; KNN: K-Nearest Neighbor; SVM: Support Vector Machine; NB: Naïve Bayes; LDA: Linear Discriminant Analysis; CHSQ: Chi square score; WLCX: Wilcoxon; NCA: Neighborhood Component Analysis; infFS: Infinite Feature Selection

Supplementary Table S8: **Significant differences in the performance (0.632+ bootstrap AUC of 1000 repetitions) between the NASNetLarge, handcrafted radiomics features and clinical factors for GTVn regression prediction.**

Features	Machine learning	Feature selection	p value
NASNetLarge vs. Handcrafted radiomics features	RF	CHSQ	0.055
	RF	WLCX	0.243
	RF	NCA	0.130
	RF	ReliefF	0.021
	RF	infFS	0.272
	KNN	CHSQ	0.006
	KNN	WLCX	0.013
	KNN	NCA	0.006
	KNN	ReliefF	0.037
	KNN	infFS	0.006
	SVM	CHSQ	0.308
	SVM	WLCX	0.480
	SVM	NCA	0.120
	SVM	ReliefF	0.029
	SVM	infFS	0.321
	NB	CHSQ	0.127
	NB	WLCX	0.051
	NB	NCA	< 0.001
	NB	ReliefF	0.010
	NB	infFS	0.094
	LDA	CHSQ	0.115
	LDA	WLCX	0.103
	LDA	NCA	0.002
	LDA	ReliefF	0.320
	LDA	infFS	0.068
NASNetLarge vs. Clinical factors	RF	CHSQ	0.165
	RF	WLCX	0.492
	RF	NCA	0.491
	RF	ReliefF	0.094
	RF	infFS	0.359
	KNN	CHSQ	0.243
	KNN	WLCX	0.351
	KNN	NCA	0.235
	KNN	ReliefF	0.074
	KNN	infFS	0.433

SVM	CHSQ	0.224
SVM	WLCX	0.049
SVM	NCA	0.448
SVM	ReliefF	0.075
SVM	infFS	0.439
NB	CHSQ	0.271
NB	WLCX	0.238
NB	NCA	0.245
NB	ReliefF	0.067
NB	infFS	0.457
LDA	CHSQ	0.348
LDA	WLCX	0.069
LDA	NCA	0.121
LDA	ReliefF	0.380
LDA	infFS	0.134

RF: Random Forest; KNN: K-Nearest Neighbor; SVM: Support Vector Machine; NB: Naïve Bayes; LDA: Linear Discriminant Analysis; CHSQ: Chi square score; WLCX: Wilcoxon; NCA: Neighborhood Component Analysis; infFS: Infinite Feature Selection

Supplementary Table S9: **Classification performance of the InceptionResNetv2 model (ReliefF + SVM) using different ΔGTVp thresholds.**

InceptionResNetv2 model (ReliefF + SVM)	Measured		AUC	Sensitivity	Specificity
	Regression	Non-regression			
Median threshold (0.46%/treatment day)	48	48	0.82	0.77	0.70
0.8%/treatment day	40	56	0.87	0.76	0.82
1.2%/treatment day	33	63	0.84	0.73	0.79
1.6%/treatment day	31	65	0.82	0.74	0.75
2.0%/treatment day	25	71	0.84	0.76	0.77
2.4%/treatment day	20	76	0.83	0.77	0.76
2.8%/treatment day	19	77	0.84	0.77	0.77
3.2%/treatment day	17	79	0.83	0.79	0.75

SVM: Support Vector Machine

Classification of primary gross tumor volume (GTVp) regression and non-regression using 3.2%/treatment day as the threshold yielded area under the curve (AUC), sensitivity, and specificity values of 0.83, 0.79, and 0.75, respectively. Classification using the median (0.46%/treatment day) as the threshold yielded AUC, sensitivity, and specificity values of 0.82, 0.77, and 0.70, respectively. There was no significant change in the AUC when the threshold was increased. The highest sensitivity was observed using 3.2%/treatment day as the threshold.

Supplementary Table S10: **Classification performance of the NSANetLarge model (ReliefF + SVM) using different ΔGTVn thresholds.**

NASNetLarge model (ReliefF + SVM)	Measured				
	Regression	Nonregression	AUC	Sensitivity	Specificity
Median threshold (1.40%/treatment day)	40	39	0.84	0.65	0.83
1.7%/treatment day	38	41	0.85	0.73	0.79
2.1%/treatment day	33	46	0.84	0.73	0.77
2.5%/treatment day	32	47	0.83	0.71	0.77
2.9%/treatment day	27	52	0.80	0.71	0.73
3.3%/treatment day	24	55	0.80	0.70	0.74
3.7%/treatment day	19	60	0.82	0.73	0.77
4.1%/treatment day	15	64	0.83	0.74	0.78

SVM: Support Vector Machine

Classification of nodal gross tumor volume (GTVn) regression and non-regression using 4.1%/treatment day as the threshold yielded area under the curve (AUC), sensitivity, and specificity values of 0.83, 0.74, and 0.78, respectively. Classification using the median (1.4%/treatment day) as the threshold yielded AUC, sensitivity, and specificity values of 0.84, 0.65, and 0.83, respectively. There was no significant change in the AUC when the threshold was increased. The highest sensitivity was observed using 4.1%/treatment day as the threshold.

References

1 Clark, K. *et al.* The Cancer Imaging Archive (TCIA): maintaining and operating a public information repository. *Journal of digital imaging* **26**, 1045-1057 (2013).
